# Handheld Ultrasound Bladder Volume Assessment Compared to Standard Technique

**DOI:** 10.7759/cureus.64649

**Published:** 2024-07-16

**Authors:** Sanna Ho-Gotshall, Casey Wilson, Errett Jacks, Rahul Kashyap

**Affiliations:** 1 Emergency Medicine, Grand Strand Medical Center, Myrtle Beach, USA; 2 Medicine, Drexel University College of Medicine, Philadelphia, USA; 3 Global Clinical Scholars Research Training (GCSRT), Harvard Medical School, Boston, USA; 4 Research, Global Remote Research Program, St. Paul, USA; 5 Critical Care Medicine, Mayo Clinic, Rochester, USA; 6 Research, WellSpan Health, York, USA

**Keywords:** ai & robotics in healthcare, urinary retention, bedside ultrasound, urine volume, point-of-care-ultrasound, emergency ultrasound

## Abstract

Urinary retention is a common complaint encountered in the emergency department (ED). Current tools for the assessment of urinary retention are either bladder volume estimation with a bladder scanner performed by nursing staff or direct visualization and measurement via bedside ultrasound performed by an emergency physician. Newer handheld ultrasound devices such as the Butterfly iQ have been brought to the market to bring ultrasound more conveniently to the bedside. A recently released handheld auto-calculation tool produces a 3D image of the bladder and instant bladder volume measurement in milliliters. However, there is a paucity of data assessing the validity of the new Butterfly iQ at the bedside. This study sought to compare the diagnostic accuracy and rated user convenience of the nursing bladder scanner, the cart-based ultrasound machine, and the Butterfly iQ auto-bladder volume tool. ED patients were prospectively enrolled and underwent bladder measurements in a randomized, pre-determined order with each modality. Measurements were subsequently compared to the gold standard of catheterization. Cart-based ultrasound had the highest agreement to catheterization when compared to the RN scanner and the Butterfly iQ. However, the Butterfly iQ and RN scanner were both considered more convenient measurement modalities than the cart-based ultrasound. The Butterfly iQ serves as a cost-effective alternative to cart-based ultrasound while providing greater general utility compared to bladder scanners.

## Introduction

Acute urinary retention (AUR) is a common emergency department (ED) complaint affecting one in three men over the age of 80 [1]. Contemporary tools for the assessment of AUR include bladder volume estimation with a handheld bladder scanner performed by a nurse or via bedside ultrasound performed by an emergency physician. Bladder scanners are widely used and accepted; however, they may be less accurate than cart-based ultrasound measurements [2]. One recent feature of smaller handheld ultrasound devices offered is the use of "auto-tools" with the goal of quickly obtaining clinically relevant measurements with ease. Software innovations implement the power of fully automated machine learning algorithms that learn as their sample size in images scanned increases. There is still limited data on this artificial intelligence (AI), but when coupled with clinical expertise, it may become more widely applicable in a variety of practice settings [3,4]. This study aimed to assess the accuracy of auto-calculation tools such as the automated bladder scanning feature on the handheld Butterfly iQ compared to measurements obtained by cart-based ultrasound and traditional bladder scanners as well as its ease of use at the bedside.

## Materials and methods

Study design

This is a prospective study performed within the Grand Strand Medical Center system located in Myrtle Beach, South Carolina. The main campus is a level 1 trauma center as well as a comprehensive stroke and cardiac center. There are two additional free-standing EDs associated with this network in which data was gathered. Attending faculty physicians and resident physicians (PGY-1-PGY-3) were the operators, all of whom completed or are participating in an Accreditation Council for Graduate Medical Education (ACGME)-affiliated residency program. All operators were trained in bladder ultrasonography by ultrasound faculty under direct supervision and given a 30-minute didactic session on the topic. Additionally, each operator had completed the American College of Emergency Physicians (ACEP) minimum requirement of 25 bladder ultrasounds in order to enroll patients in the study. The ultrasound devices used in the comparison were the GE Venue, BladderScan Prime, and Butterfly iQ.

Sample size and data collection

This study included a convenience sample of a total of 21 patients including males and females over the age of 18 presenting to the ED with a complaint of AUR. Participants were enrolled and consented for inclusion in the study over a five-month period. Patient variables recorded included age decade, gender, chief complaint, time since ED triage, as well as the amount of time they have been unable to urinate. Bladder volume was subsequently measured using the Butterfly iQ, cart-based ultrasound, and nursing bladder scanner in immediate succession, the order of which was previously randomized. After measurements were obtained, urinary catheterization was immediately performed as the gold standard bladder volume calculation, and total bladder volumes were recorded after five minutes of drainage. Operators using the three imaging devices were then asked to rate the efficiency of use on a set numeric scale ranging from 1 to 5, with 1 being the least efficient and 5 being the most efficient. In answering this question, they were asked to reflect on the ease or difficulty of workflow in obtaining measurements, satisfaction with image quality, and physical spatial considerations at the bedside. Physicians participating in the study rating ease and efficiency of use were urged to leave familiarity with individual imaging devices out of consideration when giving their rating.

Statistical analysis

Analysis was conducted on de-identified data for each measurement modality. The Bland-Altman analysis for repeated measures was conducted between (1) RN scanner and catheterization, (2) cart-based ultrasound and catheterization, and (3) Butterfly iQ and catheterization. The mean difference between bladder catheterization and each measurement modality was calculated. This was subsequently plotted against the average of the two measurements and recorded on a Bland-Altman scatter plot to assess agreement between each measurement modality against the gold standard of bladder catheterization.

The device operators were also asked to assess the ease of use and efficiency of each modality via a Likert scale of 1 through 5, with a score of 5 signifying the most efficient and user-friendly measurement tool. Once values for each modality were recorded, mean scores were calculated for each measurement modality to determine which device was perceived to be the most convenient to use in a busy clinical environment.

## Results

A total of 21 patients consented; all 21 patients completed the study with all three device measurements obtained. The majority (71%, n=15) of patients were male with a mean age of 69. The mean catheter urine volume found was 777.25 ml. The Bland-Altman analysis, as depicted in Table 1, demonstrated that cart-based ultrasound had the highest agreement to the gold standard at 0.83 when compared to the RN scanner (0.76) and the Butterfly iQ (0.65). When comparing the mean difference between bladder volume measurements via catheterization, the RN scanner was found to have the lowest mean difference at 48.38 (p=0.18). The mean difference between bladder catheterization and cart-based ultrasound was -108.17 (p=0.18), while the mean difference between bladder catheterization and Butterfly iQ was 131.29 (p=0.023).

**Table 1:** Agreement between RN scanner, cart-based ultrasound, and Butterfly iQ versus catheterization

Modality	Mean difference (mL)	Standard error (mL)	Correlation	P-value
RN scanner	48.38	57.01	0.76	0.18
Cart-based ultrasound	-108.17	60.17	0.83	0.18
Butterfly iQ	131.29	64.37	0.65	0.023

Operator surveys found that the RN scanner and Butterfly iQ were the most efficient and easy to use with a mean survey score of 3.76 and 3.76, respectively. Cart-based ultrasound was rated at 3.14 and found to be less efficient than the other two modalities (Table 2).

**Table 2:** Efficiency of use for RN scanner, cart-based ultrasound, and Butterfly iQ

Modality	Mean score (1-5)
RN scanner	3.76
Cart-based ultrasound	3.14
Butterfly iQ	3.76

## Discussion

AI has exploded in medical science and education, boasting convenience and superiority but with very little scientific validation. There is a growing list of medical image analysis AI applications with the United States Food and Drug Administration (USFDA) that range in clinical needs from detecting arrhythmia on smartwatches to triaging radiology imaging studies for critical results. Companies like GE HealthCare have reportedly invested a total of $44 million in grant money as of Fall 2023 for further development of AI-assisted ultrasound technology [5]. In 2022, the global market for bladder scanners as a product alone was estimated to be valued at USD 141.70 million [6]. These numbers reflect continued growing interest and research involved at the crossroads of AI and ultrasound.

This study aimed to evaluate the diagnostic accuracy of the handheld Butterfly iQ auto-bladder volume tool against currently available conventional methods. The bladder scanner and cart-based ultrasound had the highest agreement with the gold standard of catheterization and therefore were more accurate than the auto-tool in measuring bladder volumes. Although the RN scanner and cart-based ultrasound showed a lower difference in bladder volume measurements compared to the gold standard, these did not show statistical significance. The difference in bladder volume measurement when using the Butterfly iQ compared to catheterization was significantly different. This finding suggests that the Butterfly iQ auto-bladder volume tool may provide an overestimation of bladder volumes, which may only be clinically significant if a patient does not have AUR as it may affect treatment decisions should it be falsely positive.

Conversely, a study of smaller enrollment size with similar study parameters comparing conventional bladder scanners and 3D ultrasound devices demonstrated that the accuracy of bladder scanners was greater than cart-based ultrasound. This study found both modalities to underestimate bladder volumes on average [7]. A more recent study demonstrated in the ICU setting that cart-based ultrasound and bladder scanner both provide accurate measurements of bladder volume without statistical difference [8]. Individual mL estimates are probably less important when evaluating for acute retention, but direct bladder visualization by the treating physician may provide clinically useful information. One study discussed the use of traditional bladder scanners in which residual bladder volumes did not allow for distinction between that and other collections of fluid in the pelvis or lower abdomen. In this study, three cases were described in which cystic ovarian pathology presented initially as elevated post-void residuals resulting in catheterization that did not yield amounts equal to those interpreted on bladder scanners [9]. This is just one advantage that ultrasound devices bring to the bedside. Detecting intraluminal masses and the presence of clots or bladder stones could be missed with a conventional bladder scanner which fails to directly visualize anatomic structures. Bladder scanners can also occasionally misinterpret intra-abdominal free fluid or aneurysms as the urinary bladder and lack capabilities such as color and pulse-waved Doppler resulting in more detrimental outcomes.

When comparing ease of use, the bladder scanner and Butterfly iQ were considered most efficient through mean scores calculated from ratings obtained on a 5-point Likert scale. The Butterfly iQ is a feasible, cost-effective, and economical alternative with robust applications in underserved communities and austere settings [10]. While the Butterfly iQ auto-calculation function for bladder volume did not perform with superior accuracy, in a setting with fewer resources, this is a very reasonable alternative. When resources are limited, the Butterfly iQ performs well having a single probe for multiple functions, small size, low cost, and good image quality for most clinical applications. Disadvantages, all of which were deemed surmountable, were the large probe footprint, lower (although adequate) cardiac imaging quality, frequent overheating, and reliance on internet-based cloud storage [11]. The Butterfly iQ has also been gaining attention in the prehospital setting with considerations for emergency medical services (EMS) crews. It has been proposed that it may have a role in the prehospital setting for cardiac assessment during advanced cardiac life support (ACLS) and allow for assistance with difficult access. Its use by EMS has also been described during needle thoracostomy demonstrating increased accuracy and safe placement versus physical landmarks alone [12].

AI's relationship with ultrasound thus far has primarily served to cut down on repetitive tasks and help with quality assurance in real time such as automatically identifying high-quality image acquisition [13]. In an imaging modality relying on manual operation with significant operator dependence, AI has the potential to help physicians obtain more accurate and repeatable outcomes while using point-of-care ultrasound (POCUS). Other novel applications for AI in POCUS include standard plane recognition and organ identification, extraction of standard clinical planes from 3D volumes, and real-time guidance of image acquisitions performed by humans or robots [14]. The future of AI and ultrasound will likely combine both a physician's clinical experience and advancing technological capabilities to provide more efficient and accurate results than either alone [15].

The next steps for further investigation include conducting a similar study with a population that incorporates additional control over parameters of variability seen in this study such as blood clots or hematuria. Comparing additional handheld portable ultrasound devices and their auto-calculation features may provide further information on their accuracy and their scope of clinical uses as additional devices continue to receive updates and are brought to the bedside. Lastly, this study design represents a simplistic approach for comparing ultrasound measurement to a quantifiable gold standard collection that could be repeated with other types of procedures and applications in the ED.

Study limitations

There were several limitations to this study, primarily of which is a small sample size of data from a single institution which was heavily distributed towards male patients. Additionally, the total recorded bladder volume drained at five minutes could have varied with the non-standardization of Foley catheter sizes. Direct visualization also led to unexpected findings; in one patient, a blood clot was found in the bladder causing acute outlet obstruction. 30 mL of saline was used to displace the blood clot to allow for urinary drainage, and this may have skewed the results by causing a 30 mL increase in bladder volume measurement obtained through the Foley catheter.

## Conclusions

The auto-tool used for bladder volume estimates had less agreement with total bladder volumes than traditionally available modalities in our study. While it occasionally caused an overestimation of bladder volumes, these were not clinically relevant, and overall, the Butterfly iQ performed well in determining AUR. This handheld device was rated to be convenient, allowed for direct bladder visualization, and has tools such as color Doppler that are not available on a traditional bladder scanner.

## References

[1] Emberton M, Anson K (1999). Acute urinary retention in men: an age old problem. BMJ.

[2] Prentice DM, Sona C, Wessman BT, Ablordeppey EA, Isakow W, Arroyo C, Schallom M (2018). Discrepancies in measuring bladder volumes with bedside ultrasound and bladder scanning in the intensive care unit: a pilot study. J Intensive Care Soc.

[3] Girard E, Sankaranarayanan S (2020). Butterfly iQ auto bladder volume: development and validation of a deep learning algorithm for calculating bladder volume. http://5e42cd66afca5121212d7b69_2020.

[4] Komatsu M, Sakai A, Dozen A (2021). Towards clinical application of artificial intelligence in ultrasound imaging. Biomedicines.

[5] (2023). GE HealthCare gets $44 mln grant to develop AI-assisted ultrasound tech. https://www.reuters.com/business/healthcare-pharmaceuticals/ge-healthcare-gets-44-mln-grant-develop-ai-assisted-ultrasound-tech-2023-09-18/.

[6] (2023). Bladder scanners market. https://straitsresearch.com/report/bladder-scanners-market.

[7] Vinod NN, Nagle AS, Naimi HA (2019). Bladder volume correction factors measured with 3D ultrasound and BladderScan. Can J Urol.

[8] Schallom M, Prentice D, Sona C, Vyers K, Arroyo C, Wessman B, Ablordeppey E (2020). Accuracy of measuring bladder volumes with ultrasound and bladder scanning. Am J Crit Care.

[9] Cooperberg MR, Chambers SK, Rutherford TJ, Foster HE Jr (2000). Cystic pelvic pathology presenting as falsely elevated post-void residual urine measured by portable ultrasound bladder scanning: report of 3 cases and review of the literature. Urology.

[10] Mariani G, Kasznia-Brown J, Paez D (2017). Improving women's health in low-income and middle-income countries. Part II: the needs of diagnostic imaging. Nucl Med Commun.

[11] Burleson SL, Swanson JF, Shufflebarger EF (2020). Evaluation of a novel handheld point-of-care ultrasound device in an African emergency department. Ultrasound J.

[12] Dewar ZE, Ko S, Rogers C, Oropallo A, Augustine A, Pamula A, Berry CL (2023). Prehospital portable ultrasound for safe and accurate prehospital needle thoracostomy: a pilot educational study. Ultrasound J.

[13] Drukker L, Noble JA, Papageorghiou AT (2020). Introduction to artificial intelligence in ultrasound imaging in obstetrics and gynecology. Ultrasound Obstet Gynecol.

[14] Tenajas R, Miraut D, Illana CI, Alonso-Gonzalez R, Arias-Valcayo F, Herraiz JL (2023). Recent advances in artificial intelligence-assisted ultrasound scanning. Appl Sci.

[15] Kuang M, Hu HT, Li W, Chen SL, Lu XZ (2021). Articles that use artificial intelligence for ultrasound: a reader's guide. Front Oncol.

